# Nutritional Status in Children with Celiac Disease and Type 1 Diabetes Mellitus—A Narrative Review

**DOI:** 10.3390/nu17040728

**Published:** 2025-02-19

**Authors:** Daniela Pop, Edita Gabriela Ichim, Dorin Farcău

**Affiliations:** 1 Third Pediatric Discipline, “Iuliu Hațieganu” University of Medicine and Pharmacy, 400217 Cluj-Napoca, Romania; 2 Third Pediatric Department, Emergency Hospital for Children, 400217 Cluj-Napoca, Romania; 3 Nursing Discipline, “Iuliu Hațieganu” University of Medicine and Pharmacy, 400217 Cluj-Napoca, Romania

**Keywords:** children, celiac disease, diabetes mellitus

## Abstract

In children with type I diabetes mellitus (T1DM) and celiac disease (CD), malabsorption could add to a deficient nutritional status, resulting in impaired growth and weight and height deficits, as well as deficiencies in vitamins and micronutrients. This narrative review aims to assess the current evidence regarding the consequences of the concomitant diagnoses of CD and T1DM on the nutritional status of children. Results regarding the influence of CD and T1DM weight, height, and BMI are controversial, especially if we consider that most of the studies have a small number of patients and that adherence to a gluten-free diet is not adequately assessed. There is a lack of studies considering specific dietary habits and ethnic and cultural differences. Children with T1DM and longer time with positive serology for CD have lower levels of ferritin, vitamin D 25OH, and folate and also lower bone mineral density.

## 1. Introduction

Type I diabetes mellitus (T1DM) and celiac disease (CD) are two autoimmune diseases that frequently co-occur in children [1]. A recent meta-analysis of CD in T1DM reviewed 106 articles, including patients in 40 countries, and reported a pooled seroprevalence of CD of 9% (95% confidence interval 8–10%). In this meta-analysis, the confirmed CD prevalence was 6% (95% CI 5–7%) [2]. There was a higher prevalence in women and children [2].

In CD, the autoimmune process targets the small bowel mucosa, and in T1DM, it targets the pancreatic β islet cells [3]. Both CD and T1DM have high-risk human leucocyte antigen (HLA) genotypes [3]. DR4-DQ8 (DRB1*04-DQA1*03:01-DQB1*03:02) and DR3-DQ2 (DRB1*03-DQA1*05:01 DQB1*02:01) are common HLA haplotypes with increased risk for both CD and T1DM [3,4,5]. In 35 children with T1DM and symptoms suggestive of CD included in the study of Siddiqui et al., DQ2/DQ8 was found in 34%, DQ2 in 31.4%, and DQ8 in 25% [6]. Genes involved in T- and B-cell activation or maturation and cytokine genes commonly associated with the two autoimmune diseases have also been described but with less consistent evidence [7].

The pathogenesis of both T1DM and CD is an interplay between genetic and immunological factors, viral infections, diet, and the microbiome [3]. For CD, the antigen responsible for triggering the autoimmune response is gluten [3]. After gluten crosses the epithelium of the small bowel, gliadin is deaminated by tissue transglutaminase [3]. It binds to DQ2 or DQ8 on antigen-presenting cells, triggering an inflammatory response of the CD4^+^ T cells by producing inflammatory cytokines [3,8]. For T1DM, the possible antigens are insulin or proinsulin, glutamic acid decarboxylase, and insulinoma antigen-2. Pancreatic islets are infiltrated with CD4^+^CD8^+^T cells, a stage called insulitis [3]. In time, this leads to the apoptosis of β cells [7], the production of antibodies against the triggering antigens, and ultimately, hyperglycemia. In children with both T1DM and CD, studies have found alterations in intestinal permeability and heightened local immunoregulatory mechanisms [9,10]. Blood cytokine balance suffers significant changes, with important elevation of cytokines like IL-5, IL-8, IL-13, IL-15, IL-17F, IL-22, IL-27, IP-10, MIP-1*β*, sIL-2R*α*, sTNFRII, and TNF*α* in this category of children, in particular, compared with children with CD without T1DM [10,11,12]. Increased serum levels of these cytokines were correlated with the grade of mucosal damage assessed according to the Marsh classification [12].

In the long term, in adult patients, a diagnosis of CD and T1DM for more than 10 years increases the risk for the development of diabetic retinopathy [13]. In adults with diabetes and undetected CD, the prevalence of retinopathy and neuropathy is higher [14,15]. In young patients with diabetes and CD, non-compliance to a gluten-free diet was associated with increased albumin excretion rate [16]. However, young adults with T1DM and CD have a similar nutritional status and bone metabolism as healthy controls [17]. Not only does a gluten-free diet positively impact the nutritional status of patients with both T1DM and CD, but long-term complications like macro- and microvascular complications might be prevented [18].

Introducing additional dietary restrictions can be challenging for both patients and their parents. A gluten-free diet is less palatable and implies excluding valuable sources of vitamins B1, B2, and iron, which are found in wheat, rye, and barley [19]. The association of the two autoimmune diseases could have consequences on controlling the level of blood glucose but also on growth, weight gain, levels of albumin, ferritin, vitamin D, bone mineral density, or long-term malignancy risks.

## 2. Aim

This narrative review aims to assess the current evidence regarding the consequences of the concomitant diagnoses of CD and T1DM on the nutritional status of children. The questions that drove this research were as follows: How is the growth in weight and height of children with T1DM impacted by the associated CD? Are there any consequences of the association of the two diseases on the absorption of certain minerals and bone mineral density? A literature search was conducted in Scopus, Google Scholar, PubMed, Web of Science, and Cochrane Library for studies in English on children (aged 0 to 18 years) diagnosed with T1DM and CD. The search terms that were used are “celiac disease”, “diabetes mellitus”, “children”, and “gluten-free diet”. This review analyzed studies reporting weight, body mass index (BMI), and height of children diagnosed with T1DM and CD.

## 3. Studies Evaluating Weight, Height, and BMI in Children with T1DM and CD

Screening patients with T1DM for CD is especially important because only up to 10% of the patients develop digestive symptoms, with most experiencing mild symptoms or being asymptomatic [18]. Children under the age of 4 years are more likely to develop both T1DM and CD than those aged more than 9 years, and girls have a higher risk than boys [20]. In this study from Italy, in which more centers from all over the country were involved, a significant number of children diagnosed with T1M and CD were included [20]. The diagnosis of T1DM preceded the diagnosis of CD in 88.4% of the patients, with 12% being diagnosed simultaneously with the two diseases [20]. Over the years, anti-gliadin, endomysium, and anti-tissue transglutaminase antibodies have been used to screen patients with CD.

Impaired weight gain and short stature would suggest CD, but some studies show that these are not affected at the moment of the diagnosis of CD in an important number of patients. Characteristics of the studies included in this analysis are summarized in Table 1. A case–control study by Hansen et al. compared the data of 106 children with T1DM with the same number of healthy children [21]. Eleven patients from the diabetic group were diagnosed with CD, with two prior to this study and nine after. One of the patients diagnosed with both T1DM and CD was a patient with Down syndrome who was excluded from the calculations of height, weight, and BMI [21]. The study found a significantly younger age at diagnosis of T1DM in the group of patients associated with CD compared with patients who had only a diagnosis of T1DM, and the authors hypothesized a more aggressive autoimmune process in these children [21]. The median age at diagnosis of diabetes was 3.2 years in the CD+T1DM children and 7.4 years in children with T1DM without CD [21]. Height was significantly lower in children with T1DM and CD, but there were no differences in BMI and weight compared with children with T1DM without CD [21].

An Italian study by Barera et al. prospectively followed children with T1DM over 6 years [22]. During this time, they found 17 children who were also diagnosed with CD. Screening for patients with CD was conducted with the determination of endomysium antibodies, and confirmation was based on a small bowel biopsy histopathology exam [22]. One patient was diagnosed with CD before the beginning of the study, so the analysis included 16 children with both T1DM and CD [22]. The score for BMI standard deviation (SD) did not differ in patients with or without CD, but there was a significant improvement in this score after a gluten-free diet in children with CD [22].

Saukkonen et al. retrospectively evaluated the data of 776 children with T1DM. Screening tests for gliadin and/or reticulin antibodies were performed [23]. In 18 patients, CD was confirmed based on small bowel biopsy results [23]. There were no changes in SD scores for height before or after the diagnosis of CD [23]. The authors noted a significant increase in weight for height after the diagnosis of CD [23].

Similar results were reported by Amin et al. in 11 children diagnosed with T1DM and CD for the SD scores for height, with no changes found compared with controls (children with T1DM) [24]. This was a longitudinal study, with available data before the diagnosis of CD [24]. Mean SD for BMI and weight were significantly lower in children with T1DM and CD at diagnosis of T1DM and diagnosis of CD [24]. Twelve months after introducing a gluten-free diet, there was a significant increase in BMI SD scores [24].

In a long-term evaluation of children by Crone et al., no significant differences were noted in height and weight between endomysium antibody-positive and -negative diabetic children [25]. In this study, a small group of children also diagnosed with CD based on small bowel biopsy results showed no significant difference in these parameters before or one year after the gluten-free diet was started [25].

Sanchez-Albisua et al. found that 62 patients (22.1%) of 281 patients diagnosed with T1DM were positive for CD serology (antigliadin and endomysium antibodies) [26]. Out of the twelve patients in whom intestinal biopsy was recommended, CD was confirmed in nine patients [26]. Three patients complained of gastrointestinal symptoms consistent with CD, and four had iron deficiency anemia [26]. The authors report a significant increase in height standard deviation after introducing a gluten-free diet [26].

A large cohort of children (19796) diagnosed with T1DM was included in the study of Kaspers et al. [27]. They divided the cohort into three study groups: Group 1 children with only T1DM, Group 2 children with TIDM and positive serology for CD (antigliadin, endomysium, or tissue transglutaminase antibodies) and no small bowel biopsy, and Group 3 children with T1DM and CD diagnosed based on small biopsy changes [27]. In only 13 patients, CD was diagnosed before T1DM [27]. The study found that weight, height, and BMI SD scores were significantly reduced in children with both T1DM and CD compared to children with only T1DM [27]. In the long term, the height difference increased compared to children with only T1DM, with a loss of 0.8 SD score 9 years after the onset of diabetes [27]. The height difference was primarily noticed in children younger than 11 years at the onset of diabetes, a group of patients who already had a delay in height at the onset of T1DM [27].

Saadah et al. retrospectively collected data on 21 children diagnosed with T1DM and CD [28]. They matched each child with both T1DM and CD for age, sex, and duration of diabetes, with two children diagnosed only with T1DM (42 children) [28]. The authors report that twenty of the patients (95%) with T1DM and CD complained of at least one symptom suggestive of a digestive disorder before the diagnosis of CD [28], unlike other studies [29] in which only one patient had classic symptoms of CD). The patients were screened with serological tests (antigliadin antibodies in all patients and endomysium antibodies in 16) [28]. The mean weight for age and BMI z-scores increased significantly after 12 months. The mean height for age z-score also increased after 12 months, but there was no significant difference compared to baseline values [28]. In the control group (children diagnosed only with T1DM), the weight and height for age values were higher than those measured in children associated with CD, but not significantly [28]. The weight, height, and BMI of the children diagnosed only with T1DM did not change significantly after 12 months [28]. Symptoms improved in only 50% of the patients despite good dietary adherence [28]. Even though symptoms did not improve in some patients, their gain improved after the gluten-free diet, and the authors mentioned the “silent” effect of CD [28].

Analysis of children with T1DM with positive endomysium antibodies showed no difference in SD scores for height and BMI compared with children diagnosed only with T1DM and negative serology for CD [30]. These children had an earlier onset of diabetes compared with children with negative serology for CD. Of 98 children with T1DM and positive serology for CD, 74 children had histological changes consistent with silent CD (Marsh 3 and 4) [30]. BMI SD scores increased significantly in these children during follow-up after the recommendations for a gluten-free diet were made in both boys and girls [30]. On the other hand, the z-scores for height decreased significantly [30]. Of the 74 children who were recommended a gluten-free diet, 25 were noncompliant [30]. There was a trend for lower BMI SD scores for these patients, but this trend did not reach statistical significance. A similar trend was not recorded for height z-scores [30].

A much higher number of patients with T1DM reported symptoms suggestive of CD in the study of Hansen et al. (almost 85%) [31]. In children with positive antigliadin, endomysium, and/or tissue transglutaminase antibodies, the diagnosis of CD was confirmed with a small bowel biopsy [31]. Patients with CD had a significantly lower SD score for height and weight [31]. Patients were followed for two years, having significant weight increase, but the height increase was not statistically significant [31].

More than half of the children (8/15) diagnosed with CD and T1DM reported by Poulain et al. were diagnosed with T1DM before the age of 4 years [32]. Growth was normal for age in all these patients [32]. After the gluten-free diet, neither height nor BMI increased significantly [32].

A large multicenter study that included 411 children diagnosed with T1DM and CD (confirmed by histology of the small bowel) compared anthropometric parameters with those recorded in children without CD [33]. SD scores for weight and height were significantly lower in children with both autoimmune diseases, but BMI SD scores were not different [33]. After one year of follow-up of 183 patients with both T1DM and CD, there was a slightly lower SD score for both weight and height in children with both autoimmune diseases [33]. This difference increased and became statistically significant after 4 more years for both parameters [33].

Another case–control study assessed 30 diabetic children positive for tissue transglutaminase antibody, comparing them with 30 seronegative children with T1DM [34]. Eleven of the seropositive children had small bowel biopsies, with nine of them showing histologic features of CD [34]. In patients with severe villous atrophy, significantly lower weight, height, and BMI values were found [34]. Patients with partial villous atrophy had normal body weight and height [34]. The four patients diagnosed in this study with severe villous atrophy followed a strict gluten-free diet and, after 1–2 years, had a significant increase in height and weight z-scores [34].

Narula et al. also reported a significantly younger age at diagnosis of diabetes among patients with T1DM and CD [35]. In this study, 76.4% of the patients reported more than one gastrointestinal symptom [35]. With the gluten-free diet, symptoms resolved, and there was a significant improvement in weight and BMI z-scores [35]. The authors point out the importance of targeted questions regarding symptoms suggestive of CD and that some patients recognized their symptoms after improvement with a gluten-free diet [35].

Simmons et al. compared nutritional parameters in children with T1DM with and without positive serology [36]. In this study, tissue transglutaminase antibodies were determined, and based on the results, 71 children were included in the positive group and 63 in the negative one [36]. The two groups were matched for age, sex, and diabetes duration. The study found lower values of the z-scores for weight, BMI, and midarm circumference in children with positive serology [36]. Small bowel biopsy results confirmed CD in 35 of the 71 diabetic children (50%) with positive serology [36]. The authors further compared the group of children with confirmed CD and T1DM with a group of diabetic children with negative serology, matched for age, sex, and diabetes duration. BMI z-scores were significantly lower in children with both autoimmune diseases [36]. There were no significant differences in the scores for height between the two groups [36]. After 2 years of follow-up, children with positive biopsy (Marsh score 3) had higher z-scores for height [37].

In the study published by Sun et al., patients with T1DM and CD were compared with a similar number of patients (49) diagnosed with only T1DM matched for age at diagnosis and duration of diabetes [38]. Screening tests (antigliadin, endomysium, and tissue transglutaminase antibodies) were performed on diabetic patients in the clinics where they were followed [38]. Small bowel biopsy confirmed the diagnosis in all patients included in the CD group [38]. Height, weight, and BMI were not impaired before the start of the gluten-free diet [38]. There were no significant changes regarding these parameters at 1 and 2 years after a gluten-free diet [38].

Abid et al. collected the data from 468 children diagnosed with T1DM, finding 33 children with positive serology for CD (positive antigliadin and endomysium antibodies until 2002 and then tissue transglutaminase and endomysium antibodies) [39]. Small bowel biopsy confirmed the diagnosis in twenty-three children, of which one child did not complete the study [39]. Eleven children out of twenty-two (50%) with T1DM and CD entering the study had symptoms suggestive of CD [39]. Height, weight, and BMI were recorded before and after introducing a gluten-free diet [39]. No statistically significant changes were found between the recorded values [39]. Before introducing the gluten-free diet, values of the SD for height, weight, and BMI showed no impairment in children associating T1DM and CD [39].

The study of Gopee et al. aimed to compare albuminuria in children with only diabetes with children with diabetes and CD [40]. The latter group had lower levels of albuminuria and a slower rate of progression of albumin excretion [40]. In 24 children diagnosed with T1DM and CD with a gluten-free diet, the authors found no differences in height and BMI when compared with children with only T1DM [40]. The authors hypothesized that CD or gluten-free diet confers a degree of renal protection [40].

Mackinder et al. selected 23 out of 45 children diagnosed with both T1DM and CD who had no other chronic diseases and had available anthropometrics 2 years before and 2 years after the diagnosis of CD [41]. They were compared with a control group of patients with CD (n = 23) and a control group of children diagnosed with T1DM (n = 44), matched for age, sex, and age at diagnosis with the study group [41]. Among other parameters, height, weight, and BMI z-scores were also compared to the UK 1990 reference data [41]. For children with both T1DM and CD, these parameters were not significantly different at any point of the study as compared to the UK 1990 reference data or with the control groups, including children diagnosed with either CD or T1DM alone [41]. The authors concluded that the association of CD and T1DM does not affect children’s nutritional status and growth [41].

Westman et al. compared 20 children (15 females) diagnosed with both CD (confirmed with small bowel biopsy) and T1DM with a control group of children (40 participants) matched for age, sex, and duration of diabetes [42]. Children with both T1DM and CD were recommended a gluten-free diet, but only 30% of them followed a strict diet. The study assessed the dietary intake of the patients, finding lower values only in protein and sodium for children diagnosed with CD [42]. Statistical analysis found no significant differences in macronutrients, micronutrients, and energy intake [42]. The study found no differences in z-scores for height, weight, and BMI [42]. Another study by Acerini et al. on a small group of patients associating the two autoimmune disorders reports no significant differences in z-scores for BMI and height after 2 years of a gluten-free diet [29]. However, after the start of the diet, there was an increase in BMI’s SD score [29].

More recent studies on large numbers of patients, with data extracted from registries for children with T1DM from Germany, the UK, Austria, and Australia, report lower scores for SD for height in patients associated with CD [43]. The authors underline the rising incidence of CD in non-white ethnic groups in populations considered to have a lower risk for this disease [43].

In a US-based multi-institutional study, Simmons et al. suggested that there are different consequences of CD on boys and girls in children with T1DM [44]. Girls, although they followed a gluten-free diet, were shorter. Preschool girls had a lower z-score for weight but no differences in middle childhood [44]. In boys, weight z-scores were constantly lower throughout childhood and adolescence [44]. In children with silent CD and T1DM screened with endomysium antibodies and tissue transglutaminase antibodies and confirmed as celiac patients with small bowel biopsy changes, SD scores for BMI were not different from those of diabetic-only controls, neither before nor one year after a gluten-free diet was recommended [45].

In a randomized controlled trial, Kaur et al. evaluated anthropometric parameters in patients (both children and adults) with silent CD and T1DM with or without a gluten-free diet [46]. For the gluten-free diet group, there was a significant increase in weight at 6 and 12 months and in BMI at 12 months [46]. Söderström et al. assessed the association between compliance with a gluten-free diet and, among other factors, BMI in children with T1DM and CD [47]. Of the 60 patients entering the study, 19 were diagnosed with CD prior to T1DM [47]. The authors found a compliance of 68%, lower than in other reports of children with only CD [47]. They found no significant correlation between compliance and BMI in children with T1DM and CD, but there was a trend of higher BMI in those who were compliant with a gluten-free diet [47].

**Table 1:** Studies that included evaluation of weight, height, and BMI in children with CD and T1DM.

Authors	Year of Publication	Type of Study	Number of Children with CD and T1DM Included in the Study Group	Age at Diagnosing T1DM (Years)	Age at Diagnosing CD (Years)	Duration Between Diagnosing T1DM and Diagnosing CD (years)	Method of Diagnosing CD
Hansen et al. [21]	2001	Case–control	11	3.2 (median) 0.7–9.3 (age range)	10.8 (median) 2–16 (range)	3.9 (median) 1–9 (range)	Small bowel biopsy
Barera et al. [22]	2002	Prospective Longitudinal	17	7.88 ± 5.69 (mean ± SD)	-	-	Small bowel biopsy
Saukkonen et al. [23]	2002	Retrospective	18	8 ± 4.5 (mean ± SD)	8.8 ± 8.9 (mean ± SD)	-	Small bowel biopsy
Amin et al. [24]	2002	Case–control	11	-	13.8 (median) 2.6–17.3 (age range) 11.2 (mean)	4.2 (0.9–7.2) median (range) 3.8 (mean)	Small bowel biopsy
Sanchez-Albisua et al. [26]	2004	Retrospective	9	6 (mean)	10.7 (mean)	4.7	Small bowel biopsy
Kaspers et al. [27]	2004	Prospective Case–control	127	5.8 ± 4 (mean ± SD)	12.2 ± 4.6 (mean ± SD)	4.3 ± 3.8 (mean ± SD)	Small bowel biopsy
Saadah et al. [28]	2004	Retrospective Case–control	21	4 ± 2.7 (mean ± SD) 0.9–9.9 (age range)	7.5 ± 3 (mean ± SD) (1.6–12.9) (age range)	-	Small bowel biopsy
Rami et al. [30]	2005	Case–control Longitudinal	98	6.5 ± 4.1 (age at manifestation of DM)	10.0 ± 5.4	-	98 cases with positive serology 74 confirmed with small bowel biopsy
Hansen et al. [31]	2006	Case–control	33	5.8 (mean) 0.7–12.7 (age range)	-	-	Small bowel biopsy
Poulain et al. [32]	2007	Retrospective	15	6 ± 4.2 (mean ± SD) 1.1–14.1 (age range)	9.4 ± 4.8 (mean ± SD) 0.9–16 (age range)	-	Small bowel biopsy
Sun et al. [38]	2009	Case–control Longitudinal	49	5.9 ± 4.1 (mean ± SD)	9.1 ± 3.7 (mean ± SD)	3.2 ± 2.8 (mean ± SD)	Small bowel biopsy
Narula et al. [35]	2009	Retrospective	17	3.8 (median) 1.4–11 (age range)	9.5 (median) 4.2–16.6 (age range)	-	Small bowel biopsy
Abid et al. [39]	2011	Retrospective Longitudinal	22	6.8 (mean) 1.1–13.2 (age range)	11.1 (mean) 4.3–17.7 (age range)	4.2 (mean) 0.3–13.6 (age range)	Small bowel biopsy
Frohlich-Reiterer et al. [33]	2011	Retrospective Longitudinal	411	5.9 (mean)	-	7.7 (mean)	Small bowel biopsy
Mackinder et al. [41]	2014	Retrospective Case–control	23	5.3 ± 3.4 (mean ± SD)	10.7 ± 2.8 (mean ± SD)	5.4 ± 3.2 (mean ± SD)	Small bowel biopsy
Simmons et al. [44]	2017	Retrospective Case–control Longitudinal	215	5 (median)	9 ± 4 (mean ± SD)	-	135 small bowel biopsy-proven
Craig et al. [43]	2017	Prospective	1835	5.4 (median) 2.9 ± 8.7 (range)	Median age 8.1 years (5.3–11.2 years)	-	Small bowel biopsy
Berioli et al. [45]	2019	Case–control Longitudinal	16	7.97 (mean) 1–16 (range)	11.31 (mean)	3.16 years (mean) 2–6 (range)	Small bowel biopsy
Söderström et al. [47]	2021	Retrospective Longitudinal	60	8.87 (mean)	-	-	Small bowel biopsy

Conflicting results can be observed when summarizing the main findings of these studies. While some authors [21,27,31,33,34,43] report a lower height of children with T1DM and CD, others found no difference compared to children with T1DM [23,24,25,30,32,36,38,39,41,42]. The height difference was mainly noted in children with younger ages at diagnosis of diabetes [21,27].

Some studies reported lower BMI SD scores in children with T1DM and CD [24,27,31,33,34,36], while others found no significant difference compared to controls [21,22,30,38,39,41,42].

On the other hand, there are reports of increased weight [22,23,24,28,30,31,34,35,46] and height [26,28,34,37] after a gluten-free diet was recommended. Some studies found no impact of gluten-free diet on weight [25,29,32,38,39,40,41,42,45] or height [23,25,29,31,32,38,39,40,41,42], while others report a decrease in height [30,33] or weight [33] of children with T1DM and CD after they started a gluten-free diet.

Analyzing the impact of a gluten-free diet on metabolic control in children with T1DM is beyond the purpose of our review. However, since some of the studies we analyzed followed the patients’ nutritional status for a longer or shorter time after CD was diagnosed and a gluten-free diet was recommended, we mentioned the changes they noted in the anthropometric parameters after this change in the children’s diet.

A gluten-free diet could change the clinical picture of patients with CD and T1DM and impact the glucose level, insulin requirements, and lipid profile. Healing of the intestinal mucosa improves absorption and could lead to a high BMI and imbalance of glycemic control [48,49]. Gluten-free products have a high glycemic index, are rich in saturated fat, and are low in proteins and fibers [48]. A recent systematic review analyzed current evidence of the impact of a gluten-free diet on growth, glycemic control, lipid metabolism, and quality of life of children with T1DM and CD [49]. The review included moderate-high-quality studies considering adherence to a gluten-free diet as a key parameter in interpreting the effects of this diet. The authors concluded that growth was similar in children with T1DM and CD to that of children with only T1DM, and there were no differences between patients with T1DM and CD and T1DM only in glycosylated hemoglobin (HbA1c) [50] or total daily insulin dose [49]. Only a few studies evaluated the effect of a gluten-free diet on post-prandial glucose, lipid profile, and quality of life of children with T1DM and CD. Based on these limited data, the reviews’ authors reported positive effects on these parameters [49,51].

On the other hand, some authors questioned the benefits of a gluten-free diet in children with silent CD [19]. In this study, Castellaneta et al. found that levels of tissue transglutaminase decreased in 40% of the children diagnosed with T1DM and became negative in 20% without a gluten-free diet [19]. The authors support the hypothesis of a temporary serologic positivity for CD and advocate postponing small bowel biopsy and a gluten-free diet [19].

## 4. Micronutrients and Vitamin Levels in Children with T1DM and CD

The late diagnosis of CD might cause delays in development, the burden of adding gluten-free products to an already difficult diet, and the cost of expensive gluten-free products. Good glycemic control must be ensured, but sometimes gluten-free products are high in fat and sugar content and low in micronutrients [47].

Barera et al. found decreased ferritin concentration in patients with both CD and T1DM as opposed to patients diagnosed only with T1DM [22]. Serum folate, albumin, and ferritin levels did not change significantly in children with CD and T1DM after a gluten-free diet compared with the values recorded before this diet was recommended [22]. Serum ferritin and folate levels were normal in only one patient in Acerini et al.’s study [29]. No statistically significant changes were observed after 2 years of a gluten-free diet [29]. Poulain et al. recorded low ferritin levels in one patient out of fifteen diagnosed with CD and T1DM [32]. One patient had low levels of vitamin K, one had low levels of vitamin A, and another one had low levels of phosphorus [32].

Vitamin D 25OH and B12 levels were significantly higher in children with T1DM and CD but within normal ranges [36]. Ferritin levels did not differ between the two groups [36]. There were also no differences in bone density between diabetic children with CD and those without [36]. The same group of researchers followed this group of patients for 2 years [37]. Forty-three children with T1DM and positive serology for CD were on a gluten-free diet, and thirty-six had a regular diet [37]. Fifteen of the seventy-nine (19%) diabetic children with positive tissue transglutaminase (ten on a gluten-free diet) antibodies continued to have high levels of antibodies after 1 year, and nine of them after 2 years of follow-up [37]. Compared to the children in the negative serology group, those who remained positive had significantly lower ferritin levels, vitamin D 25OH, and bone mineral density [37]. After 2 years of follow-up, the biopsy-positive group of patients had lower levels of ferritin [37].

In 2016, Simmons et al. published a study assessing bone mineral density in children with T1DM and positive tissue transglutaminase antibodies in a larger cohort [52]. They concluded that children with both autoimmune diseases had significantly lower bone mineral density, suspecting a negative synergistic effect of CD and inadequate glycemic control on bone mineralization [52].

For patients (both adults and children) with CD and T1DM on a gluten-free diet, there were no differences from the same category of patients but rather on a regular diet for calcium, phosphorus, alkaline phosphatase, parathormone, or bone mineral content at 6 and 12 months of follow-up [46].

Hansen et al. found a significant increase in hemoglobin, mean corpuscular volume, and ferritin after 2 years of the recommended gluten-free diet in CD and T1DM children [31]. The folate levels also increased significantly at 6, 12, and 18 months of follow-up, but a statistically significant increase was not reached at 24 months for this parameter [31].

Comparing the levels of folic acid in adult patients with T1DM and CD, CD, T1DM, and healthy controls, Nunes-Silva et al. found significantly lower levels in patients with one or both autoimmune disorders before and after starting a gluten-free diet [17]. This can be caused by malabsorption before the healing of the intestinal mucosa. However, after gluten was withdrawn from the child’s diet, the low levels of folic acid were explained by the low folate content of the gluten-free products [17]. This might explain low levels of vitamins and other minerals in children who follow a gluten-free diet [17].

In the Artz et al. study, bone mineral density and apparent density were lower in diabetic patients with severe villous atrophy [34]. These parameters were also significantly reduced in patients with partial villous atrophy [34]. A gluten-free diet resulted in an increased trend in bone mineral density [34].

## 5. Current Study Limitations and Future Research

Results regarding the influence of CD and T1DM on weight, height, and BMI are controversial, mainly because most studies have a small number of children, including those who lack data and poorly compliant patients. There is a lack of studies considering specific dietary habits and ethnic and cultural differences. Methods of diagnosing CD evolved, so new studies assessing nutritional status in children diagnosed with CD and T1DM with the more feasible serologic test are needed. One of the limitations of multicenter studies that were published until now is that serology analysis and histopathology exams for CD were performed in different laboratories. Prospective studies are needed to assess vitamin and micronutrient levels before and after a gluten-free diet was recommended for both symptomatic and asymptomatic patients, considering the new criteria for diagnosing CD and distinguishing between early and late diagnosis of CD in diabetic children. The content of micronutrients and vitamins in gluten-free products must also be considered and, if necessary, improved. Future studies must also assess thoroughly the adherence to the gluten-free diet of the patients entering the study.

## 6. Conclusions

Diagnostic criteria for CD have changed over the years. Recommendations regarding screening for CD are 20–25 years old. Some older studies only report data from patients diagnosed with CD based on biopsy results, excluding patients with positive serology from the analysis but with normal biopsy results.

The absence of symptoms suggestive of CD in children with T1DM highlights the importance of screening in atypical, silent, or latent forms, as most of the time, the diagnosis of T1DM precedes the diagnosis of CD. Children who are eventually diagnosed with both T1DM and CD have a younger age at diagnosis of T1DM, making them more likely to have a delay in growth and weight. Current studies show conflicting data in anthropometric parameters in children with T1DM and CD, both before and after a gluten-free diet is recommended. Most of them do not adequately evaluate adherence to this diet. A younger age at the onset of diabetes might be correlated with lower height in children who are also diagnosed later with CD, compared with patients with only T1DM. Some studies report low ferritin, folic acid, vitamin levels, and bone mineral density in children with T1DM and CD.

## Abbreviations

The following abbreviations are used in this manuscript.

T1DM	type I diabetes mellitus
CD	celiac disease
BMI	body mass index
SD	standard deviation
HbA1c	glycosylated hemoglobin
